# Pesticide toxicity assessment and geographic information system (GIS) application in small-scale rice farming operations, Thailand

**DOI:** 10.1038/s41598-021-04523-x

**Published:** 2022-01-11

**Authors:** Ekarat Sombatsawat, Dana Boyd Barr, Parinya Panuwet, Mark Gregory Robson, Wattasit Siriwong

**Affiliations:** 1 College of Public Health Sciences, Chulalongkorn University, Bangkok, Thailand; 2 Gangarosa Department of Environmental Health, Rollins School of Public Health, Emory University, Atlanta, GA USA; 3 School of Environmental and Biological Sciences, Rutgers University, New Brunswick, NJ USA

**Keywords:** Biomarkers, Health occupations

## Abstract

The objectives of the study were to evaluate the impact of pesticide exposure on farmer health during non-active rice farming and active rice farming periods and present the change in the individual cholinesterase activities (%reduction) on the geographic information system (GIS) mapping in Nakhon Ratchasima Province, Thailand. Acetyl- and butyryl-cholinesterase (AChE and BuChE) activities were monitored during both study periods using Test-mate ChE (Model 400). The location of paddy fields was specified using Garmin geographic positioning system MAP 62s. Fifty-eight farmers who participated in this study had an average age of 49.2 ± 6.9 years. Higher prevalence of all health symptoms was observed among farmer participants during the active rice farming period comparing to the non-active rice farming period (*p* < 0.01). Furthermore, farmers had significantly lower activities of AChE and BuChE during the active rice farming period comparing to the non-active rice farming period (*p* < 0.01). Our findings indicate that the GIS mapping indicate that the cases with a significant enzyme inhibition have dispersed across the agricultural and the nearby residential areas. This, investigation can be used to promote safer use of pesticides among farmers and mitigate pesticide exposure among residents living in close proximity to a rice field.

## Introduction

Pesticides are widely used in the agricultural sector to protect plants from insects and increase productivity. Globally, farmers applied several pesticides, including insecticides, herbicides, fungicides, rodenticides, and others, at a rate of about 3.5 million tons per year^1^. Typically, farmers can be exposed to pesticides through all three routes (i.e. inhalation, ingestion or dermal contact) with dermal contact tending to dominate the other routes. Occupational pesticide exposures have been linked to multiple short- and long-term adverse effects on human health, even exposure occurring at low levels^2^. In developing countries, health burdens relating to pesticide exposures have been increasing owing to their high volume and frequency of pesticide use as well as easy accessibility^3^. Pesticide poisoning is a common phenomenon in developing countries. Many cases of poisoning go unreported; therefore, pesticide exposures constitute a more serious health threat than recognized^4^. Improper handling of pesticides contributes greatly to severe acute poisoning and chronic health effects^5^ on multiple systems such as respiratory, endocrine, gastrointestinal, neurological, and reproductive systems^2,6^.

The most common types of pesticides used in crop protection are highly toxic humans^7^. Thailand is ranked 18th globally in pesticide use, with 35,287 tons per annum of pesticide usage^8^. Prior reports indicated that the morbidity rate of pesticide intoxication was 76.4–96.6 per 100,000 people, and the number of hospitalized patients was estimated to be approximately 49,000–61,000 each year^9^. Upon entering the human body, organophosphates (OPs) and carbamates exert their primary toxicity through the inhibition of acetylcholinesterase (AChE) and butyrylcholinesterase (BuChE) activities, which are essential enzymes for numerous functions of cholinergic pathways in the central nervous systems^10^. AChE enzyme is present in human red blood cells (RBCs), nervous tissues and skeletal muscles, while BuChE is synthesized in the liver present in serum, and has its only known purpose as an exposure “sink”^4^. Inhibition of AChE and BuChE causes an over-accumulation of the neurotransmitter acetylcholine at cholinergic nerve synapses, resulting in an acute cholinergic syndrome via continuous neurotransmission^11^. A change in cholinesterase enzyme activity can be used as a biomarker of exposure and effect for OP and carbamate pesticide exposure and may be used to inform adverse clinical health problems.

The long-term health effects may arise from residential exposure to large-scale pesticide applications in their vicinity^12^. A prior study demonstrated that breast cancer risks were significantly associated with residents living in a 1-mile radius of organophosphate pesticide disposing sites^13^. A geographic information system (GIS) used in conjunction with global positioning systems (GPSs) can enable the verification of pesticide spraying locations^14^. It also uses to track and map cases during a disease outbreak or a specific hazard event. This recent technology can also provide new opportunities to collect ecological data on agricultural pesticide exposure and visualize a pesticide poisoning pattern within a specific area. In this study, we evaluated the impact of pesticide exposure on farmer health and used GIS mapping to overlay the change in cholinesterase activities observed from farmers during the active rice farming period on farming area in Phimai District, Nakhon Ratchasima Province, Thailand. Using this information, health protective guidelines for reducing pesticide exposure among farmers and those who live near farms can be developed.

## Results

### Demographic characteristics

The demographic and farm activity characteristics of the participants are summarized in Table 1. Half of the farmers were male (50%), and the age range was 35–59 years (Mean 49.2 ± 6.87 year). The majority of the farmers completed high school (74.1%) and self-reported having never smoked or consumed alcohol (86.2% and 74.1%, respectively). Insecticides were used by more than half of the participants (53.5%), followed by herbicides (31%) and others pesticides (15.5%). Approximately 84.5% of the participants sprayed pesticides using a motorized backpack sprayer. About half of the participants (53.5%) reported applying pesticides for a period of greater than 2 h per application.

**Table 1:** Demographic and farm activities characteristics.

Parameters	n (%)
**Age**	
18–35 years	16 (27.58)
36–59 years	42 (72.42)
Mean (± SD) = 49.2 (± 6.87), min–max = 24–59 years	
**Sex**	
Male	29 (50.00)
Female	29 (50.00)
**Education level**	
Has not been to school	2 (3.45)
Elementary school	1 (1.72)
High school	43 (74.14)
College or university	12 (20.69)
**Smoking status**	
No	50 (86.21)
Yes	8 (13.79)
**Alcohol consumption**	
No	43 (74.14)
Yes	15 (25.86)
**Type of pesticide**	
Insecticide (Chlorpyrifos, Malathion, Carbofuran, Methomyl etc.)	31 (53.46)
Herbicide (Glyphosate, 2, 4-D, Paraquat, etc.)	18 (31.03)
Other (Metaldehyde, Zinc phosphide, Biochemicals etc.)	9 (15.51)
**Type of sprayer**	
Manual backpack sprayer	9 (15.51)
Motorized backpack sprayer	49 (84.49)
**Spraying duration**	
Less than or equal 2 h/time	27 (46.55)
More than 2 h/time	31 (53.45)
Mean (± SD) = 2.64 (± 1.12), min–max = 0.5–6.0 h	

### Pesticides handling practices

Table 2 shows that most of the farmer participants (60.4%) read the label of pesticide products before using them and the majority (51.7%) used the recommended amounts of pesticides. Approximately 79% of the participants took a shower with soap immediately after pesticide application. More than half of the participants (65.5%) kept pesticide products at home. About 41% of the participants washed the equipment after pesticide application. All farmers wore long fabric pants, and almost all of them (98.3%) wore long sleeve fabric shirts while handling and applying pesticides. The vast majority of the participants used hats (84.5%), shoes (74.1%), and masks (67.2%). Nearly half of farmers (46.6%) reported wearing gloves and a few of them (1.7%) reported wearing eye protection.

**Table 2:** Pesticides handling practices.

Pesticides handling practices			AChE		*p*-value	BuChE		*p*-value
			No depression n (%)	Depression n (%)		No depression n (%)	Depression n (%)	
Read the label of pesticides products before application	No	23 (39.65)	8 (13.79)	15 (25.86)	0.060	9 (15.51)	14 (24.14)	0.778
	Yes	35 (60.35)	21 (36.21)	14 (24.14)		15 (25.87)	20 (34.48)	
Used the recommended amounts of pesticides	No	28 (48.27)	10 (17.24)	18 (31.03)	0.036*	10 (17.24)	18 (31.03)	0.397
	Yes	30 (51.73)	19 (32.76)	11 (18.97)		14 (24.14)	16 (27.59)	
Took a shower with soap immediately after pesticides application	No	12 (20.69)	4 (6.90)	8 (13.79)	0.195	7 (12.07)	5 (8.62)	0.181
	Yes	46 (79.31)	25 (43.10)	21 (36.21)		17 (29.31)	29 (50.00)	
Washed the equipment after pesticides application	No	34 (58.62)	11 (18.97)	23 (39.65)	0.001*	11 (18.97)	23 (39.65)	0.097
	Yes	24 (41.38)	18 (31.03)	6 (10.35)		13 (22.41)	11 (18.97)	
Kept pesticide products at home	No	20 (34.49)	14 (24.14)	6 (10.35)	0.027*	6 (10.35)	14 (24.14)	0.202
	Yes	38 (65.51)	15 (25.86)	23 (39.65)		18 (31.03)	20 (34.48)	
**Used personal protective equipment (PPE)**								
Hat	No	9 (15.52)	2 (3.45)	7 (12.07)	0.288	2 (3.45)	7 (12.07)	0.204
	Yes	49 (84.48)	27 (46.55)	22 (37.93)		22 (37.93)	27 (46.55)	
Mask	No	19 (32.76)	11 (18.97)	8 (13.79)	0.038*	6 (10.35)	13 (22.41)	0.092
	Yes	39 (67.24)	18 (31.03)	21 (36.21)		18 (31.03)	21 (36.21)	
Long sleeve shirt	No	1 (1.72)	1 (1.72)	0 (0.00)	0.313	0 (0.0)	1 (1.72)	0.397
	Yes	57 (98.28)	28 (48.28)	29 (50.00)		24 (41.38.)	33 (56.90)	
Long pants	No	0 (0.00)	0 (0.00)	0 (0.00)	NC	0 (0.00)	0 (0.00)	NC
	Yes	58 (100.00)	29 (50.00)	29 (50.00)		24 (41.38)	34 (58.62)	
Gloves	No	31 (53.45)	12 (20.69)	19 (32.76)	0.065	15 (25.86)	16 (27.59)	0.246
	Yes	27 (46.55)	17 (29.31)	10 (17.24)		9 (15.51)	18 (31.03)	
Shoes	No	15 (25.86)	8 (13.79)	7 (12.07)	0.764	8 (13.79)	7 (12.07)	0.275
	Yes	43 (74.14)	21 (36.21)	22 (37.93)		16 (27.59)	27 (46.55)	
Eye protection	No	57 (98.28)	28 (48.28)	29 (50.00)	0.313	24 (41.38.)	33 (56.90)	0.397
	Yes	1 (1.72)	1 (1.72)	0 (0.00)		0 (0.00)	1 (1.72)	

### Cholinesterase activity during non-active rice farming and active rice farming periods

As shown in Table 3, the activities of AChE and BuChE were measured in blood samples as a biological marker of OP and carbamate exposure and effect. The average activity of AChE was 3.13 ± 0.61 U/mL (Min–max = 1.89–4.46U/mL) and 2.75 ± 0.65 U/mL (Min–max = 1.32–4.38 U/mL) as observed during the non-active rice farming (baseline) and active rice farming periods, respectively. The average activity of BuChE was 1.87 ± 0.52 U/mL (Min–max = 0.70–2.93U/mL) and 1.53 ± 0.46 U/mL (Min–max = 0.52–2.92 U/mL) during the non-active rice farming (baseline) and active rice farming periods, respectively. As expected, on average, the activities of AChE and BuChE significantly decreased during the active rice farming period, where pesticides are applied more frequently and with greater (paired simple t-test: *p* = 0.004 and *p* = 0.001, respectively). From both periods, the change in AChE and BuChE activities were calculated for each farmer and expressed as a percentage value (% change, with negative value indicated enzyme suppression). Out of 58 subjects during the active rice farming period, 29 (50%) subjects had the % AChE change of ≥ 15%, while 31 (53.5%) subjects had the % BuChE change of ≥ 15%. Table 4 summarizes the % change of AChE and BuChE activities that were observed from the farmer participants.

**Table 3:** Cholinesterase activities observed during non-active and active rice farming periods.

Biological markers	All (n%)	Mean ± SD	Range	t	*p*-value
**AChE (U/mL)**					
Non-active rice farming	58 (100.00)	3.13 ± 0.61	1.89–4.46	2.98	0.004
Active rice farming	58 (100.00)	2.75 ± 0.65	1.32–4.38		
**BuChE (U/mL)**					
Non-active rice farming	58 (100.00)	1.87 ± 0.52	0.70–2.93	3.68	0.000
Active rice farming	58 (100.00)	1.53 ± 0.46	0.52–2.92

**Table 4:** Percentage of AChE and BuChE depression levels.

Percentage of cholinesterase depression levels	AChE n (%)	BuChE n (%)
No depression (≥ 0%)	22 (37.94)	15 (25.86)
Low to moderate depression (< 0 to − 14%)	7 (12.06)	12 (20.69)
Significant depression (≥ − 15%)	29 (50.00)	31 (53.45)

In addition, as seen in Table 2, during the active rice farming period, there was a significant association between AChE depression status (depressed vs. not depressed) and pesticide application related tasks. No depression means that having a positive % change in the enzyme activities, and depression means that having a negative % change in the enzyme activities. The results showed that AChE depression was associated with “not using” the recommended amounts of pesticides (chi-square test: *p* = 0.036), washing the equipment after pesticide application (*p* = 0.001), and keeping the pesticide products at home (*p* = 0.027). Use of masks was statistically significantly associated with non AChE depression (*p* = 0.038). Linear regression analyses, controlled for age, sex, alcohol consumption (yes/no), and smoking status (yes/no), revealed that the AChE activities were weakly but significantly associated with the summed health symptom scores during the active rice farming period (r^2^ = 0.14, *p* = 030). No significant association was found between the AChE activities and the summed health symptom scores during the non-active rice farming period. No significant associations were found between the BuChE activities and the summed health symptom scores in both periods. When classified by sex, no significant associations were found between either AChE or BuChE activities and the summed health symptom scores in any periods.

### Health symptoms

**Figure 1:**
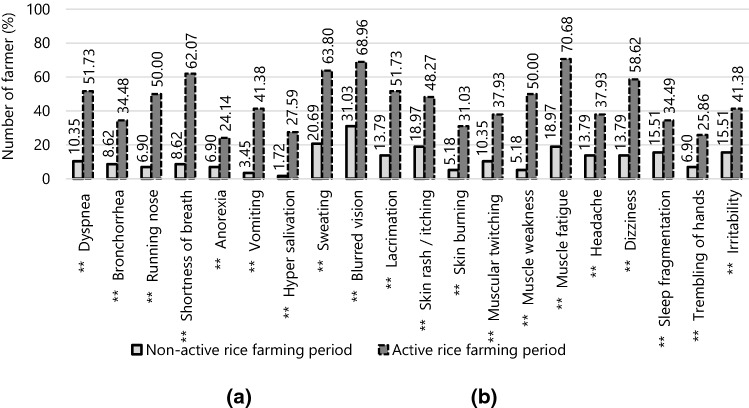
Comparison of health symptoms observed among the participants between the non-active rice farming period (**a**) and the active rice farming period (**b**).

The prevalence of specific health symptoms (n = 20) observed from both non-active rice farming and active rice farming periods are presented in Fig. 1. Just under a third of participants had health symptoms during the non-active rice farming period, while over half of them had muscle fatigue (70.7%), blurred vision (69%), sweating (63.8%), shortness of breath (62.1%), dizziness (58.6%), and dyspnea and lacrimation (51.7%) during the active rice farming period. Other symptoms including bronchorrhea, runny nose, anorexia, vomiting, hyper salivation, skin rash/itching, skin burning, muscular twitching, muscle weakness, headache, sleep fragmentation, trembling of hands, and irritability, were reported by fewer than half of the farmers. In addition, all symptoms (represented by the summed health symptom scores) were significantly increased during the active rice farming period (paired sample t-test, *p* = 0.001).

### GIS mapping

**Figure 2:**
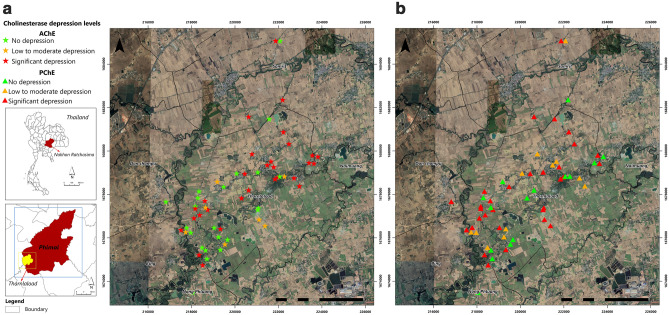
AChE (**a**) and BuChE (**b**) depression levels presented on the GIS maps during the active rice farming period. Figure was created by the ArcGIS version 10.8 software (Environmental Systems Research Institute, Inc, USA, version 10.8, http://www.esri.com).

**Figure 3:**
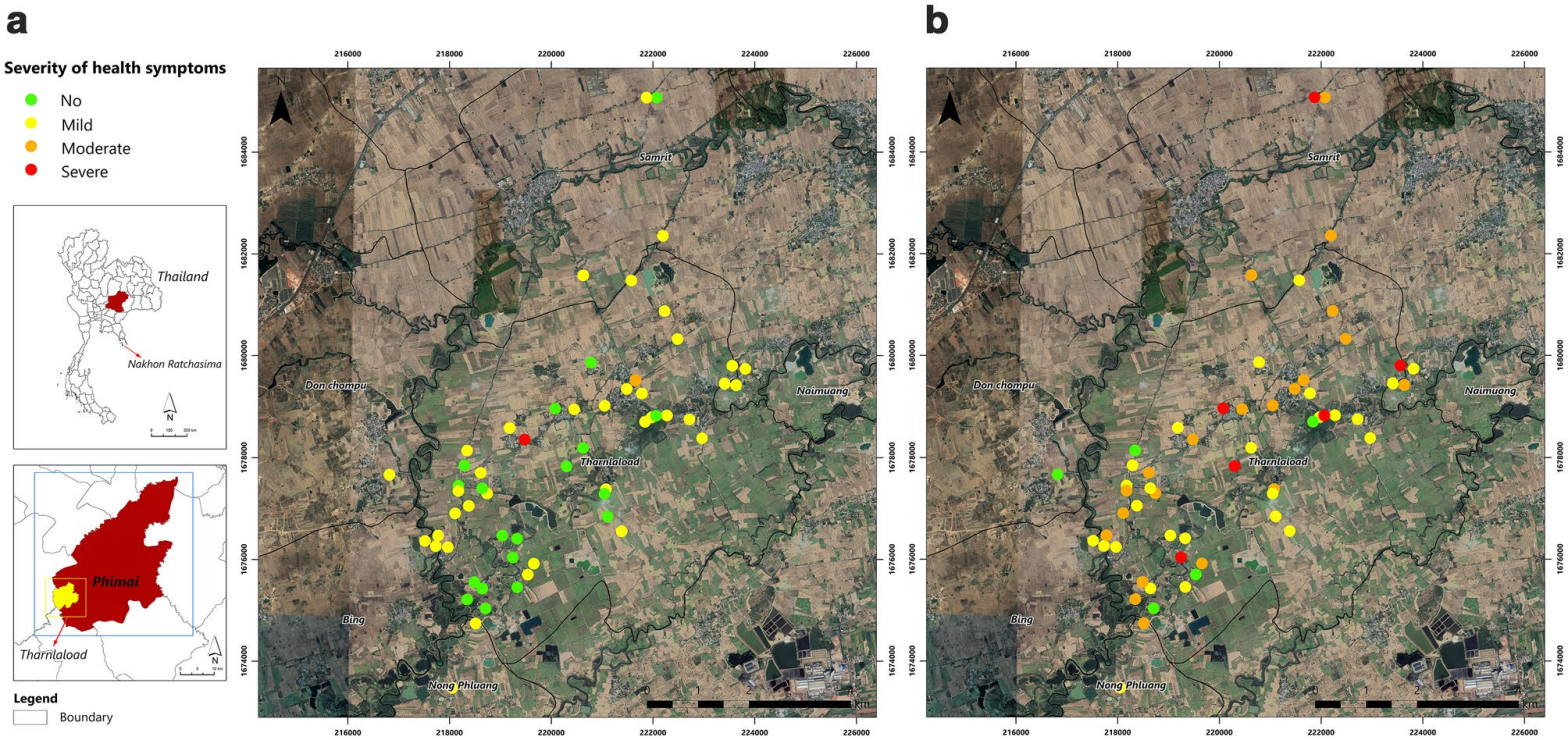
Severity of health symptoms among farmers presented on the GIS maps during both study periods. Figure was created by the ArcGIS version 10.8 software (Environmental Systems Research Institute, Inc, USA, version 10.8, http://www.esri.com). (**a**) Non-active rice farming period. (**b**) Active rice farming period.

Table 4, there were a total of 29 and 31 cases with significant AChE and BuChE depression (defined as having a % change of greater than − 15%), respectively. As seen in Fig. 2, cases with significant AChE and BuChE depression were dispersed across the farming areas and continued to show up in the residential areas. No poisoning cluster was identified on the map. The severity of health symptoms among farmers, classified based on the summed health score, is shown in Fig. 3. During the non-active rice farming, 32.8% (n = 19) of farmers had no symptom, 63.8% (n = 37) had mild symptoms, 1.7% (n = 1) had moderate symptoms, and 1.7% (n = 1) had severe symptoms. Conversely, during the active rice farming period, farmers reported, (8.6%, n = 5) of farmers reported no symptom (44.83%, n = 26) had mild symptoms, (36.2%, n = 21) had moderate symptoms (10.3%, n = 6) had severe symptoms. No cluster was identified on the map based on the summed health symptom scores.

## Discussion

The present study was conducted to evaluate the farmer health effects of occupational pesticide exposure and to evaluate cholinesterase activities during non-active and active rice farming periods and to apply it to GIS mapping. The majority of farmers conducted pesticides-related activities by themselves, including preparing, mixing, loading, and spraying. In Thailand, pesticides are sold with limited or poor restrictions. Thai farmers have easy access to pesticides and they can purchase all kinds of pesticides from community retailers. Insufficient knowledge and improper behaviors regarding safe pesticide handling among farmers in developing countries are the primary reasons for the increased health effects of occupational pesticide exposure^19^. Many of the farmers failed to read the pesticide label before application^20^ hence, almost half of farmers in this study did not use the pesticides according to the recommended amounts and directions. Moreover, both washing the equipment after pesticide application and keeping pesticide products at home were significantly associated with the frequency of AChE depression. This association may derive from the fact that farmers often used bare hands to clean their equipment after use. Prior studies have indicated that para-occupation exposure is a predominant pathway for family members to be exposed to pesticides^21^. In addition, storing pesticides in the home increases the chance of being exposed to insecticide residues by family members. Various studies have reported that the improper use or minimum use of PPE is present in many low-income countries^6,20^. Even though farmers generally use some form of PPE to protect themselves from pesticide exposure, the use of PPE is normally restricted by many factors such as insufficient accessibility, financial strain, lack of practical knowledge, and physical discomfort. Our finding indicates that wearing masks can reduce the frequency of having AChE and BuChE depression during the active rice farming period when OP and carbamate exposures occurred. This finding agrees with a pervious study^1^. A prior experimental study also showed that wearing various types of respiratory protective equipment (RPE) (such as a surgical mask, sun hat, bandana, and half facepiece respirator) can protect farmers against pesticide exposure compared to no RPE use^22^. Although self-reported symptoms can be subjective, we attempted to classify the severity level of symptoms suggestive of overall pesticide toxicity. Nonetheless, the subjective health information might be beneficial to inform the future preventive stategies^23^.

Cholinesterase activity depression is used as a biomarker of OP and carbamate pesticide exposure and effect mornitoring^24^. We demonstrated small-scale rice farmers had significantly decreased activities of AChE and BuChE enzymes during the active rice farming period compared to the non-active rice farming period (baseline period). This result is in agreement with previous studies carried out among Iranian farmers^25^. We noted that some farmers had low levels of ChE activity before rice farming tasks commenced (during the non-active rice farming period) which likely resulted from take-home exposures from other family members or neighbors^26^. This may have resulted in noticeable health symptoms during this inactive rice farming activities^27^. Del Prado-Lu suggested that continual periodic use of OP and carbamate by farmers can result in longer term cumulative exposures making them more at risk for health symptoms and ChE depression after additional pesticide use events^28^. However, our study found that both AChE and BuChE were not significantly associated with health symptoms in the non-active rice farming period (pre-exposure period). So, some symptoms may result from other causes including heavy work, outdoor heat exposure, and dehydration. We did find, however, that all health symptoms indicative of pesticide poisoning were significantly increased during the pesticide application period (during the active rice farming period). Even though farmers in this study reported some PPE use, it may not be effective against inhalation and dermal contact exposures because of their composition (e.g., fabric that is not water-resistant). Neupane et al.^29^ reported that 14 of 17 symptoms identified in their study were significantly different between Nepali farmers who used pesticides and controls who did not use pesticides. In addition, the majority of farmers in this study reported that they had at least one health symptom associated with pesticide intoxication. This finding was supported by a prior study^24^.

Our findings also demonstrate the application of GIS technology for identifying a spatial pattern of individual AChE and BuChE depression after exposure to pesticides in the paddy areas during the active rice farming period. GIS can be used to record historical data on pesticide applications and to estimate exposure levels and evaluate the proximity between residential and agricultural fields^30^. The GIS application will also permit the identification of pesticide poisoning clusters when cases were collected and plotted on the GIS map. The finding will be critical for establishing and launching several preventive programs in the future. In addition, the cholinesterase depression and GIS information can be applied to identify key locations, resulting in the collection of experimental samples (such as water sources, soil sources, or more subjective variables) from a specific demographic group within strictly defined OP and carbamate exposure areas, for future health studies. Furthermore, GIS can simplify assist public health officials with planning and management of pesticide exposure reduction programs, and lead to more efficient decision-making^31^. Thus, GIS-based pesticide exposure metrics can address all of these issues through the capacity to incorporate multiple data sources with locational, dated information, specific chemicals, and hazard levels.

There are several limitations of this study that should be considered. First, the sample size of this study is small. However, the findings were similar to prior studies with more participants^17^. In addition, these subjects represent a large fraction of farmers who applied pesticide themselves in the community. Majority of Thai rice farmers do not apply pesticides themselves anymore as they hire pesticide applicators to do the task. Second, farmers might still have been exposed to pesticides while growing vegetables, chilies, and other plants for domestic consumption, or more likely from food contamination, during the non-active rice farming period resulting in lower ChE levels which could have masked significant pre- and post-exposure changes. Third, the self-reported symptoms could be subject to information bias. Fourth, ChE inhibition may not be a sensitive enough biomarker to pick up more subtle symptoms. Regardless, our study does provide useful information, especially in low-income, high pesticide-use areas. Future studies are recommended to compare the rate of ChE change within the individual shortly before and after pesticide spraying to minimize this limitation.

## Conclusions

Inappropriate OP and carbamate use and exposure prevention contribute to the significant decrease in both AChE and BuChE activities, and the increase in health symptoms among farmers. Therefore, farmers should be made aware of the safety practices of pesticide handling and application and the proper use of PPE through effective education and training programs. Importantly, the government should consider changing the current policy to allow effective restrictions of pesticide importation, production, and application. In addition, GIS can assist the assessment of agricultural pesticide exposure in the general population and can enable the location verification and pattern visualization of the OP and carbamate poisoning cases. Our work can be used to assist the establishment of a pesticide application free zone to minimize pesticide exposures in the residential areas.

## Methods

### Study area and study periods

**Figure 4:**
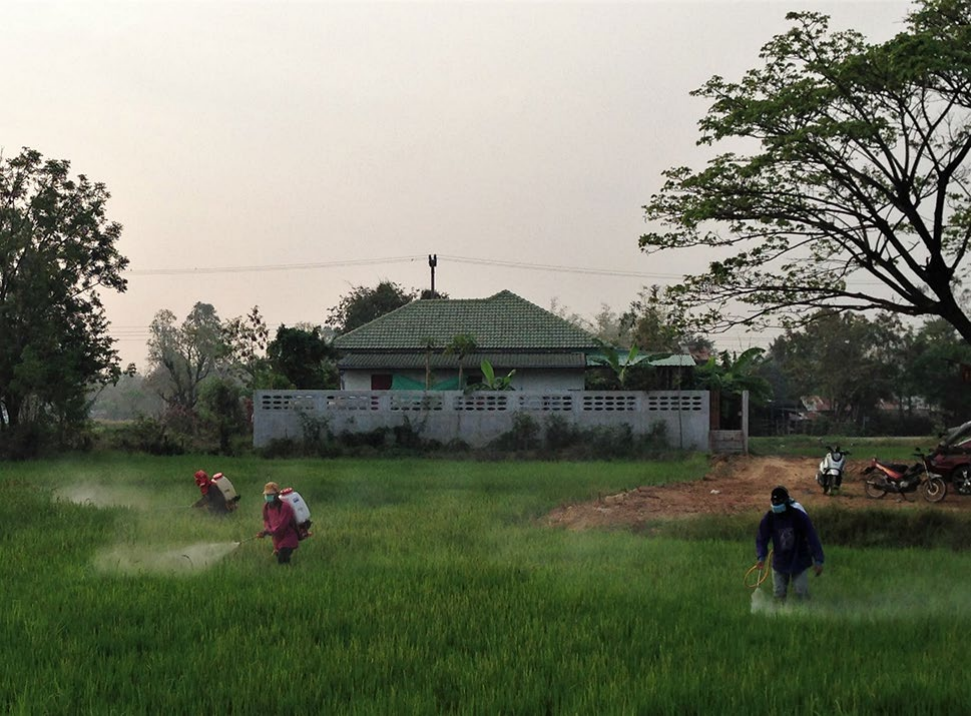
A cultivation area that is in close proximity to the residential area.

The study area was located in Phimai District, Nakhon Ratchasima Province, northeastern Thailand, within the Universal Transverse Mercator (UTM) range of East 229,949.19 and North 1,684,288.58 (a latitude of 15° 13′ 14.39′′ N and a longitude of 102° 29′ 10.24′′ E or 15.220663 and 102.486177, respectively) (Fig. 1). Pesticides and other agrochemicals have been used heavily to protect agricultural products and increase agricultural yields for a long time. These cultivation areas were typically located next to each other and in close proximity to residential areas (Fig. 4). This observational study was conducted in two periods: non-active rice farming and active rice farming periods. The non-active rice farming period was defined as a period of more than 30 days before the beginning of a rice growing season. The active rice farming period started 30 days after rice farming was commenced and it was the period when pesticide application was begun.

### Study population and questionnaire

A pretest–posttest study was conducted in small-scale rice farmers of Phimai District. A total of 75 farmers were eligible and willing to participate in this study. They were enrolled using a convenience sampling (non-probability sampling). In order to participate in the study, farmers must have met the following eligibility criteria: (1) aged ≥ 18 years, (2) directly involved with OP and carbamate pesticide application, (3) no apparent diseases related to ChE activity (e.g., cardiovascular disease, liver failure, myocardial infarction, kidney disorders, malnutrition, and drug addiction) and (4) speak Thai with no communication problems. Only 58 farmers participated and completed in all stages (pre- and post-data collection). A structured questionnaire was administered individually using a face-to-face interview by a trained interviewer. The questionnaire was designed to collect the following data: (1) demographic and farm activity characteristics, (2) pesticide handling practices, and (3) health symptoms (yes/no).

### Health symptom scores

Participants were asked about health symptoms that they experienced during non-active and active rice farming periods. Each health symptom was given a score (yes = 1 and no = 0), and all scores were summed to create the summed health symptom score variable. For the GIS application, the summed health symptom scores were also categorized into four categories: (1) no symptom, (2) mild symptoms (2nd–50th percentile, with 1–8 symptoms), (3) moderate symptoms (51th–75th percentile, with 9–14 symptom), and (4) severe symptoms (> 75th percentile, with 15–20 symptoms). The Research Institute for Health Sciences (RIHES) Experimentation Committee, Chiang Mai University, Thailand approved the study protocol (no. 8/62). All procedures were conducted following the Declaration of Helsinki and written informed consent was obtained from all participants. Moreover, if needed, individual medical consultations were provided according to discussed clinical findings.

### Determination of cholinesterase activity

The activities of AChE and BuChE were determined using the EQM-mate Cholinesterase Test System, Model 400 (EQM Research Inc., 2003). During the non-active rice farming period (a baseline period), blood samples were collected from individual participants at least 30 days before the beginning of a crop season. This interval provides enough time for the enzymes to be recovered and for the enzyme activity to return to a normal range via metabolic detoxification processes if OP and carbamate exposure occurred during the previous growing period^15^. The exposure period was defined as 30 days after the crop season started and when pesticide application was first used (the active rice farming period).

Blood specimens for this analysis were collected from cleaned finger pricks into capillary tubes (approximately 20 μL) by registered nurses. The enzyme activities were expressed as units per milliliter (U/mL). Enzyme measurements were performed at ambient temperature in the laboratory (25 °C). Some of these ChE data were presented and published in a prior study^16^. The changes in AChE and BuChE were calculated and expressed as a percentage value using the equation listed below^17,18^. The %AChE and BuChE changes were treated as a continuous variable.

1 $${\text{Percent change of AChE }} = \, \left[ {\left( {{\text{AChE non-active rice farming }}{-}{\text{ AChE active rice farming}}} \right) /{\text{ AChE non-active rice farming}}} \right] \, \times { 1}00,$$

2 $${\text{Percent change of BuChE}} = \, \left[ {\left( {{\text{BuChE non-active rice farming }}{-}{\text{ BuChE active rice farming}}} \right)/{\text{BuChE non-active rice farming}}} \right] \, \times { 1}00.$$

Based on the % change values, the AChE and BuChE depression status variables (dichotomous variable) were created based on the % change as follows: no depression (% change above 0), and depression (% change below 0). The AChE and BuChE depression percentage variables and the AChE and BuChE depression status variables were used for further statistical analyses. The AChE and BuChE depression scale variables were also created using the following criteria: no depression (% change above 0%), low to moderate depression (% change from below 0 to − 14), and significant depression (% change ≥  − 15). These variables were used for the GIS mapping to indicate the areas where OP and carbamate poisoning may occur.

### GIS map creation

Global positioning device (GPS) (GARMIN GPSMAP 62s) was used to determine the latitude and longitude coordinates of the participant farms. The ArcGIS version 10.8 software was applied to plot the paddy fields of participants. A map was created using the boundaries of Nakhon Ratchasima Province and Phimai District and Google Maps for Farm, Village, and Economic Areas in 2021. Then, the participant’s average AChE and BuChE depression levels were overlaid onto the farming map to indicate spatial characteristics or the location of the poisoning cases.

### Statistical analysis

Data were coded, keyed, and analyzed using the IBM Statistical Package for Social Sciences Licensed, V.22 (IBM Corp.). Descriptive statistics were performed on the qualitative general information of the participants and expressed as either frequency, percentage, mean ± standard deviation (± SD), or range (minimum–maximum). Continuous variables were tested for normal distribution using the Kolmogorov–Smirnov test. These variables are normally distributed; thus, parametric statistics were applied. The chi-square test was used to compare differences between dichotomous variables. Comparisons of the AChE and BuChE activities, including the summed health symptoms scores observed during the non-active and active rice farming periods were conducted using paired samples t-tests. Linear regression analyses, controlled for covariates, were used to evaluate the relationship between the AChE and BuChE activities and the summed health symptom scores. Covariates included age (in year) sex (male/female), alcohol consumption (yes/no), and smoking status (yes/no). The significance level was set at *p* < 0.05. NC is not calculated.
